# Harnessing the Therapeutic Potential of SGLT2 Inhibitors in Type 2 Diabetes: Challenges and Opportunities

**DOI:** 10.31662/jmaj.2024-0083

**Published:** 2024-06-28

**Authors:** Daisuke Yabe

**Affiliations:** 1 Department of Diabetes, Endocrinology and Nutrition, Kyoto University Graduate School of Medicine, Kyoto, Japan; 2 Yutaka Seino Distinguished Center for Diabetes Research, Kansai Electric Power Medical Research Institute, Kyoto, Japan; 3 Center for One Medicine Innovative Translational Research, Gifu University Institute for Advanced Study, Gifu, Japan

**Keywords:** SGLT2 inhibitors, older adults, type 2 diabetes, sarcopenia

In type 2 diabetes (T2D) management, sodium-glucose cotransporter 2 (SGLT2) inhibitors represent a remarkable paradigm shift, offering promising therapeutic benefits beyond mere glycemic control. Initially developed based on the natural compound phlorizin found in apple tree bark, these inhibitors have been extensively studied for their role in glucose metabolism ^(1)^. The foundational mechanism of SGLT2 inhibitors lies in their ability to block glucose reabsorption in the kidney’s proximal tubules, a process responsible for reclaiming 90% of the glucose filtered by the glomeruli ^(1)^. By inhibiting this pathway, SGLT2 inhibitors facilitate increased urinary glucose excretion, lowering plasma glucose levels without relying on insulin and promoting weight loss predominantly by reducing body fat mass ^(1)^.

Recent clinical endeavors, including several cardiovascular outcome trials, have illuminated additional benefits of SGLT2 inhibitors, especially their capacity to mitigate the risk of heart failure, chronic kidney disease (CKD), and cardiovascular diseases (CVD) among individuals with T2D ^(2), (3)^. These discoveries have propelled the international and domestic endorsement of these drugs for managing T2D accompanied by heart failure, CKD, and CVD ^(2), (3)^. However, the launch of SGLT2 inhibitors in Japan was met with concerns over severe adverse events (AEs), including fatalities, potentially attributed to improper drug utilization ^(1)^, which highlights a critical aspect of pharmacological interventions: the necessity of tailored therapies based on patient-specific characteristics.

In East Asian countries including Japan, the T2D phenotype is predominantly characterized by nonobesity and diminished insulin secretion, distinct from the obesity-linked insulin resistance observed in western populations. This distinction is crucial because it influences the safety and efficacy profiles of SGLT2 inhibitors, particularly among older T2D adults with multiple comorbidities like CKD and CVD. Research indicates that the AE incidences related to body fluid loss are notably higher in older patients, prompting a need for heightened vigilance during the initial stages of SGLT2 inhibitor therapy. Moreover, there is an emerging concern about the long-term use of SGLT2 inhibitors contributing to sarcopenia in older individuals ^(1)^. SGLT2 inhibitors force the excretion of 200-300 kcal energy in the urine every day and can diminish insulin secretion and enhance glucagon release in response to urinary glucose loss―mechanisms that may inhibit amino acid uptake in muscles and promote proteolysis ^(1)^; this can lead to muscle atrophy and escalate the progression toward sarcopenia. A significant reduction in skeletal muscle mass among younger T2D adults treated with SGLT2 inhibitors has been documented, which underscores the necessity for clinical trials to evaluate these effects in older populations.

Consequently, our EMPA-ELDERLY trial―a randomized, placebo-controlled study―demonstrated that the SGLT2 inhibitor empagliflozin effectively manages glycemia without adversely affecting muscle mass or strength in otherwise healthy older Japanese individuals with T2D ^(4)^. Subsequently, a recent observational study by Nagayama et al. explored the effects of combining SGLT2 inhibitors with DPP-4 inhibitor therapy ^(5)^, suggesting improvements in glycemic control, body mass index, and grip strength among young and old adults with T2D.

Despite these encouraging results, several critical questions persist. For instance, are there identifiable clinical characteristics predicting a decline in muscle mass and strength among older adults with T2D on long-term SGLT2 inhibitor therapy? In addition, what is the efficacy of these inhibitors in older adults with T2D who also suffer from impaired activities of daily living and cognitive defects?

As Japan grapples with an aging population―where approximately 70% of those with T2D are aged ≥65―the prescription of SGLT2 inhibitors demands a nuanced approach. Medical professionals must consider the specific nature of the disease, potential comorbidities, and the overall lifestyle of the patient before recommending these drugs.

In conclusion, while SGLT2 inhibitors hold substantial promise for transforming the therapeutic landscape of T2D―particularly in mitigating associated heart failure, cardiovascular and renal risks―their application must be judiciously tailored. Enhanced understanding and research into the complex interplay of age, comorbid conditions, and drug effects are essential to optimize outcomes and safeguard the health of the aging population.

## Article Information

### Conflicts of Interest

DY received consulting or speaker fees from Astellas Pharma Inc., Dainippon Sumitomo Pharma Co., Ltd, Eli Lilly Japan K.K., MSD K.K., Novo Nordisk Pharma Ltd., Nippon Boehringer Ingelheim Co. Ltd, Ono Pharmaceutical Co. Ltd, Taisho Pharmaceutical Co. Ltd, and Takeda Pharmaceutical Company Limited and clinically commissioned/joint research grants from Taisho Pharmaceutical Co. Ltd, Novo Nordisk Pharma Ltd, Arklay Co. Ltd, and Nippon Boehringer Ingelheim Co. Ltd.

### Author Contributions

DY contributed to the writing of the manuscript and is the guarantor of this work.
